# Motives for choice of work in healthcare and individual performance of medical doctors working in public multispecialty hospitals in Warsaw

**DOI:** 10.3389/fmed.2025.1456341

**Published:** 2025-02-06

**Authors:** Malgorzata Chmielewska, Jakub Stokwiszewski, Justyna Markowska, Tomasz Hermanowski

**Affiliations:** ^1^Pharmacy Division, Department of Forensic Pharmacy, Medical University of Warsaw, Warsaw, Poland; ^2^National Institute of Public Health – National Institute of Hygiene, Warsaw, Poland

**Keywords:** motivation, work performance, healthcare sector, hospitals, physicians

## Abstract

**Objective:**

This study explores the relationship between the motives driving people to work in healthcare and the individual work performance of public hospital doctors. The results are analyzed in terms of changes necessary to improve work performance among medical practitioners.

**Methods:**

A cross-sectional study was conducted among 249 medical doctors from 22 randomly selected inpatient departments of multidisciplinary public hospitals in Warsaw. Data were collected using self-administered structured questionnaires developed by WHO. Herzberg model as modified by the authors was used to identify key motives for choosing a job in healthcare. A chi-square test was used to analyze the correlations between the reasons for choosing to work as a medical practitioner and socio-demographic variables. The interdependence between individual performance and the main reason for choosing a career in healthcare was examined with a Kruskal-Wallis test.

**Results:**

The research results indicate a statistically significant relationship between the main motive for choosing a career in healthcare—namely, the individual motivation of hospital doctors, and individual work performance as measured by “notion that my work is valuable in itself” (*p* = 0.003) and “responsibility for outcomes” (*p* = 0.024) response categories. Doctors whose main motive for choosing the profession was to help patients are more likely to feel high satisfaction with “The notion that my work is valuable in itself” than others (42.5% vs. 27.0% for “other” and 28.3% for “Nature of the work itself”) and with “The sense of responsibility for the outcomes” (50.0% vs. 35.1% for “other” and 36.3% for “Nature of the work itself”).

**Conclusions:**

When examining factors affecting the professional performance of medical doctors, aspects related to the original main motive for choosing this profession and for working in the healthcare must not be disregarded. The distinctive features of this profession, including personal qualities, should perhaps be considered in the admission criteria for medical studies.

## 1 Introduction

Motivation as a significant predictor and driver of professional performance, became a subject of interest among researchers at the turn of the 19^th^ and 20^th^ centuries with the advent of the concept of evidence-based management (1).

Learning about management processes by analyzing human behavior and by embracing diverse personalities of the staff members are the preconditions for designing a high performance organization. Employee motivation is a kind of stimulus that drives the quality of work, which then generates outcomes (2), thus increasing individual performance at work and the overall organizational performance.

With regard to the healthcare sector, researchers (3–7) argue that physicians who are more engaged in their work achieve better treatment outcomes, gain more personal satisfaction, and generate considerable patient satisfaction as compared to physicians who do not feel motivated to work.

David Wigley argues that the HR management for medical practitioners should address both individual and external motivation (8), which improves performance and commitment at the organizational level (9). This approach has a positive effect on the behavior of medical doctors, extends their professional autonomy, and increases their involvement in ongoing changes (10). Additionally, it leads to improvements in clinical care indicators (10–12).

There is a perception that medical doctors view their profession as a mission to help people or aid in their recovery (13). Kadushin coined the term ”dedicatory ethic,” which he defined as the willingness to work with people and to influence people's lives. He further explained that by elevating the motive of “helping people,” this work becomes a vocation rather than just a profession (14). According to Hippocrates, love, the voice of the heart, attachment, and vocation are the guiding values that direct the right people to practice the medical profession. These guidelines have remained unchanged for centuries. Therefore, every medical doctor should recognize that the financial benefits associated with this profession are secondary to the notion of vocation (a calling), or the desire to help those in need (humanitarian motive). The view of the medical profession as merely a job providing healthcare services at fixed hours for specific remuneration can be seen as a betrayal of the medical ethos (15). The importance of the humanitarian goal pursued by medical doctors is deeply embedded in this profession, as opposed to the aspects such as the economic value derived from the work, such as income.

It appears unlikely that high school graduates choose to pursue the challenge of studying medicine purely by chance. Personality traits, such as interests and preferences, clearly play a significant role in shaping their career choices. Holland et al. asserts that, when choosing a career path, people are guided by the alignment between their expectations and values and the nature of their future work (16).

Each profession has its own unique set of goals and expectations (17). In the medical profession, these include making a significant impact on the immediate environment, achieving success, gaining appreciation, and enjoying prestige and recognition for the work performed (18, 19). Medical doctors strive to maintain professionalism and independence—they value self-reliance and aim to carry out their work in line with their professional values and ethics (19, 20). As a consequence, this professional group is characterized by personal responsibility, autonomy, and a high level of competence (21). Additionally, medical practitioners seek higher salaries, professional advancement for achieving positive health outcomes, and prestige. They also desire greater control over their environment and the ability to afford more life pleasures, among other aspirations (22, 23).

People who seek professional fulfillment and job satisfaction are more likely to achieve their goals more easily in more respected professions (22). According to the group theory, people often choose to become medical doctors in order to enjoy higher social recognition, which is certainly more pronounced in this field than in others (22). In the healthcare environment, hospitals are regarded as more prestigious places to work than, for example, primary care settings. Medical doctors also believe that hospitals in large cities offer more career opportunities, although hospitals in smaller cities may offer higher salaries (24).

Other work-related expectations of medical doctors include guaranteed employment, professional stability, a positive work environment, opportunities for growth and development, including upskilling options (22), as well as access to modern medical technologies, and proper organization of the healthcare services (25).

The ranking of individual needs can vary considerably. In general, all medical professionals strive to satisfy their needs for survival, security, belonging, status, recognition, self-esteem, self-fulfillment, and growth. It is important to determine the extent to which these needs are met by working in the healthcare sector (18), as low morale among healthcare professionals can negatively impact their overall performance, undermining the quality of the healthcare services and putting patients' lives and health at risk (26). On the other hand, it should be noted that the issues of underinvestment in healthcare workers, the mismatch between supply and demand for workers, poor working conditions, and demographic changes, including the aging of the healthcare workforce, turnover, and burnout, have contributed to a global shortage of healthcare workers, especially physicians. In April 2023, the WHO called for the protection and investment in human capital within healthcare by seeking new formulas for strategic investment in the training, employment, and retention of medical staff, among other initiatives (27, 28).

There is a lack of research on the original motives for choosing to work in the healthcare sector, and no studies were found that explore the potential impact of this factor on the individual performance of medical doctors (29). Therefore, the reasons for choosing to work in the healthcare sector should be investigated to determine whether these motives affect the future individual work performance of medical doctors. It is also important to examine this relationship within the context of public hospitals, as they represent the predominant organizational and legal form of institutionalized healthcare worldwide.

The following research hypothesis was formulated based on the literature:

H1: There is a relationship between the main motive for choosing to work in the healthcare sector and individual performance at work among medical doctors working in public hospitals.

## 2 Materials and methods

### 2.1 Research tools and data sampling

The research tool employed in this study was a validated World Health Organization (WHO) questionnaire, designed to examine the main motives behind choosing to work in the healthcare sector (individual motivation) and the work performance of medical doctors (individual performance). The sample size for a population of approximately 8,500 doctors from multispecialty hospitals was determined using a 95% confidence level, a 50% fraction size, and a maximum error margin of 7%. Twenty-two inpatient clinics/departments were randomly selected with proportional probability from the 415 hospital departments/clinics across 32 hospitals in Warsaw. A survey was conducted on a sample of professionally active medical practitioners (*n* = 249), representing approximately 8,500 medical doctors working in multispecialty hospitals. Respondents were randomly selected using cluster sampling, with hospital departments/clinics as the sampling frame. The survey included multispecialty hospitals and medical doctors at various stages: prior to specialty training, in the process of specialty training, and those who had completed their specialty training. Single specialty hospitals and healthcare professionals other than medical doctors were excluded from the study.

### 2.2 Conceptual model

Among the theories of motivation most widely used in management and organizational contexts, Herzberg's theory is frequently highlighted in the literature (30). Most contemporary research associated with this theory emphasizes the study of motivating factors (i) in industry, retail, or occupations with naturally high turnover; (ii) personal factors; and (iii) economic factors affecting job satisfaction (31, 32). However, the analysis of people's responses and reactions to various internal and external workplace factors—which aid in predicting interest in a particular job—is less commonly explored in the healthcare sector (32).

The main motive for choosing to work as a medical practitioner was identified through an open-ended question: “The main reason why I work in the healthcare sector;” individual performance aspects were measured using five closed-ended questions selected from the WHO questionnaire, based on the literature, including the Herzberg's theory (33, 34) concerning planning one's work, the feeling that the work is meaningful, a sense of responsibility for the outcomes, satisfaction with patients' recovery, and setting personal goals.

**Figure 1:**
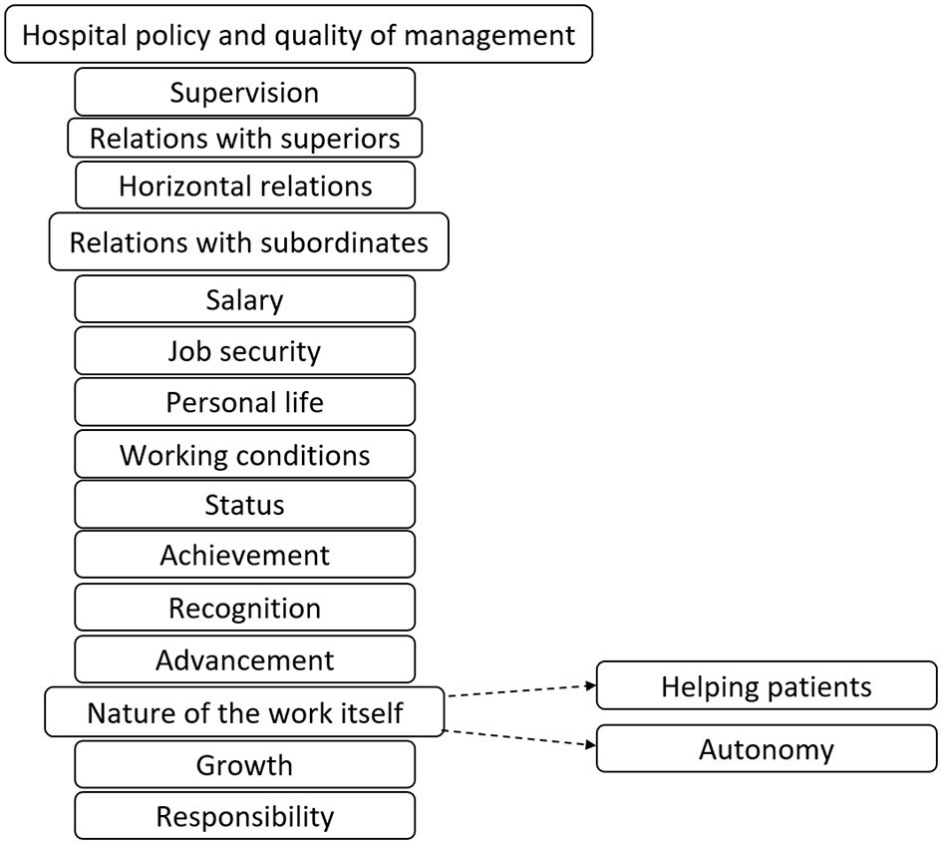
Factors behind choosing to work in healthcare—modified Herzberg model.

The open question was analyzed by the three researchers, who allocated the answers into categories based on the Herzberg model (35), namely: hospital policy and quality of management, supervision, relations with superiors, horizontal relations, relations with subordinates (vertical relations), salary, job security, personal life, working conditions, status, achievement, recognition, advancement, nature of the work itself, growth, and responsibility. Additionally, based on literature data, particularly empirical research highlighting the crucial role of motivation and commitment in healthcare professionals' overall work performance compared to other public service personnel (26), the category ”nature of the work itself” was further specified for the medical profession, unlike in Herzberg's original theory, which was based on a study of engineers and accountants (35). Specifically, two categories unique to the medical profession—“helping patients” and “autonomy” - were distinguished (Figure 1). The authors' singling out of the features specific to the medical profession is significant in that the Herzberg's theory is commonly criticized as being geared toward studying the populations of “manual workers” (36).

This means that the category “nature of the work itself” in this study includes aspects such as “the ability to perform surgeries,” “medical specialty,” “interesting work,” “interest in medicine,” “variety and sufficient challenge,” “working in a team,” and so on. The additional “helping patients” category encompassed statements reflecting the humanitarian motive for choosing to work in this profession, such as “helping sick people,” “mission,” “desire to help those who are suffering,” and “I like treating people.” Using these categories, specific trends in the responses were identified to distinguish the value of the work from the value of the goal to be achieved through the work itself.

The distribution of responses to questions about the main motive for choosing to work in the healthcare sector was also analyzed. Out of 18 analyzed categories, three categories with the most common responses (“nature of the work itself,” “helping patients,” and 16 “other” categories) were further analyzed.

### 2.3 Statistical analysis

A chi-square test was used to examine the association between the outcome variable (the reason for choosing to work as a medical practitioner) and the socio-demographic variables, which served as predictors. The questionnaire included four possible responses, on an ordinal scale, to assess the individual performance of medical doctors: high dissatisfaction, moderate dissatisfaction, moderate satisfaction, and high satisfaction. The interdependence between individual performance of medical doctors and the main reason for choosing to work in this profession was examined using the Kruskal-Wallis test. A significance level of 0.05 was adopted for this study. Data entry was performed using Epidata software version 3.1, and statistical analysis was performed using SPSS statistical package version 19.

## 3 Results

### 3.1 Motive behind choosing to work in the healthcare sector

**Figure 2:**
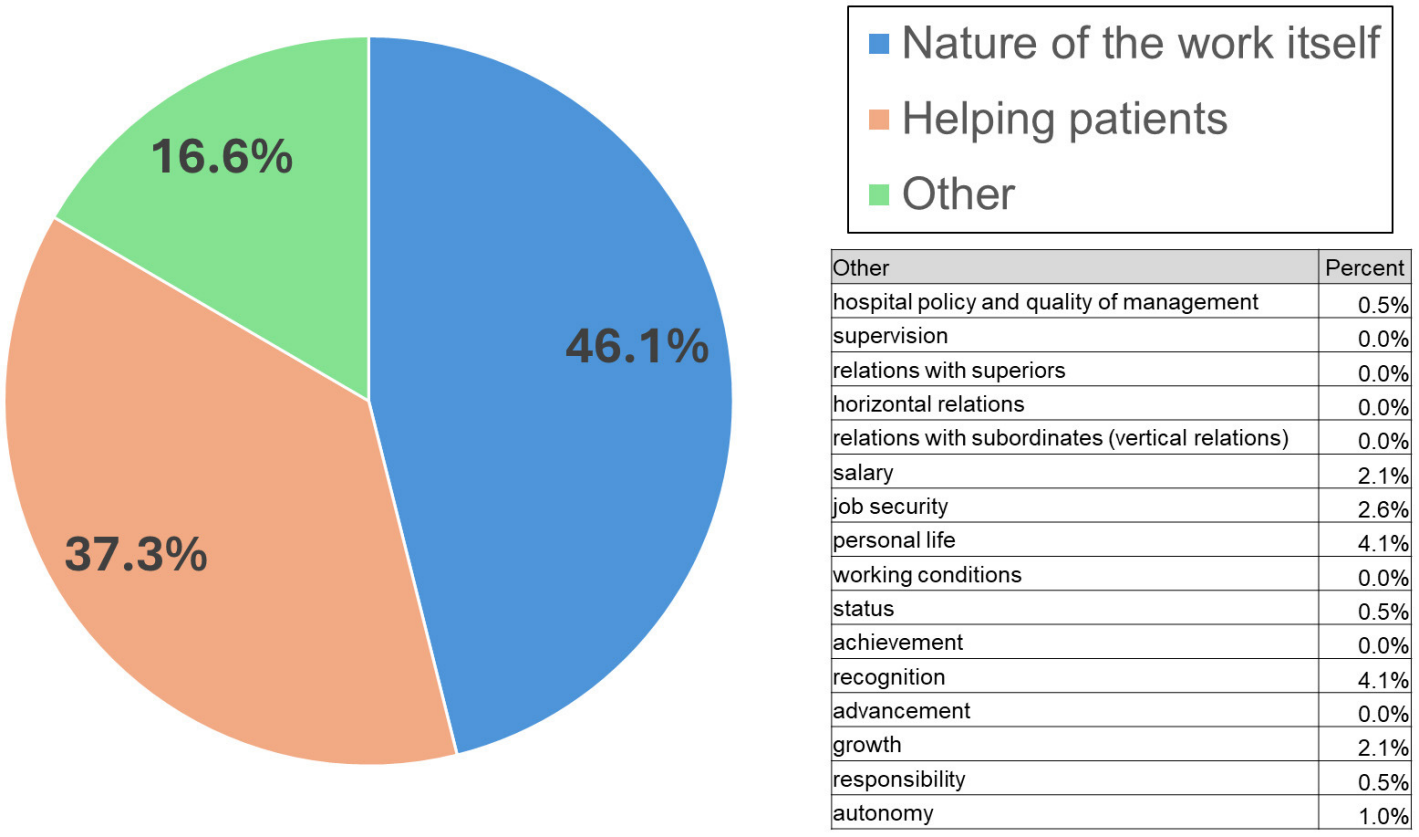
Main motive for why medical doctors choose to work in the healthcare sector.

Forty-six percent of respondents selected “nature of the work itself” as the main reason for choosing their job, while 37% of the surveyed medical doctors mentioned “helping patients” as their main motive for pursuing a career in the healthcare sector. The “other” group included 17% of people (Figure 2).

It could be helpful to pinpoint the differences between these two groups. The analyses presented in Table 1 show that men were significantly more often driven by “other” reasons in their career choices than women (as listed in Figure 2). Medical doctors specializing in surgery were significantly more motivated by “other” reasons than other specialist doctors. It is also worth noting that specialist doctors were significantly more often motivated by “other” reasons than other doctors. Doctors before specialist training were equally motivated by helping patients and the nature of the work itself, while none of them was motivated by “other” reasons.

**Table 1:** Main reason for choosing to work in the healthcare sector according to the socio-demographic characteristics.

**Socio-demographic characteristics**	**Total**		**Helping patients**		**Nature of the work itself**		**Other**		p^*^- 3 groups	p^*^Helping vs. Nature
	n^**^	**%**	***n***	**%**	***n***	**%**	***n***	**%**		
Total	193	100.0	72	37.3	89	46.1	32	16.6		
**Gender**										
Male	88	45.6	32	36.4	34	38.6	22	25		
Female	105	54.4	40	38.1	55	52.4	10	9.5	0.012	0.423
**Age**										
Up to 29	48	24.9	26	54.2	17	35.4	5	10.4		
30–39	54	28.0	18	33.3	25	46.3	11	20.4		
40–49	44	22.8	16	36.4	21	47.7	7	15.9		
50–59	28	14.5	8	28.6	14	50	6	21.4		
60+	19	9.8	4	21.1	12	63.2	3	15.8	0.244	0.106
**Residing**										
Alone	36	19.0	12	33.3	16	44.4	8	22.2		
With family	20	10.6	10	50	9	45	1	5		
With espouse/partner	133	70.4	49	36.8	62	46.6	22	16.5	0.497	0.767
**Marital status**										
Bachelor/maiden	69	38.1	31	44.9	28	40.6	10	14.5		
Married	106	58.6	36	34	51	48.1	19	17.9		
Divorced	5	2.8	0	0	5	100	0	0	0.09	0.068
Widowed (excluded from the comparison)	1	0.6	1	100	0	0	0	0		
**Providing subsistence for children**										
Yes	95	49.5	31	32.6	48	50.5	16	16.8		
No	97	50.5	41	42.3	40	41.2	16	16.5	0.351	0.148
**Providing subsistence for elderly persons**										
Yes	17	8.9	10	58.8	6	35.3	1	5.9		
No	175	91.1	62	35.4	82	46.9	31	17.7	0.134	0.138
**Providing subsistence for persons with disabilities**										
Yes	4	2.1	1	25	2	50	1	25		
No	188	97.9	71	37.8	86	45.7	31	16.5	0.837	0.837
**Employment**										
Employment contract	12	6.3	2	16.7	8	66.7	2	16.7		
Full-time position	118	61.5	39	33.1	56	47.5	23	19.5		
Residency	62	32.3	30	48.4	25	40.3	7	11.3	0.124	0.575
**Job position**										
Medical doctor prior to specialist training	12	6.2	6	50	6	50	0	0		
Medical doctor in specialty training/trainee specialist doctor	61	31.6	32	52.5	23	37.7	6	9.8		
Specialist doctor	120	62.2	34	28.3	60	50	26	21.7	0.008	0.031
**Medical specialty**										
Non-surgical	114	59.1	45	39.5	57	50	12	10.5		
Surgical	79	40.9	27	34.2	32	40.5	20	25.3	0.024	0.840

^*^p calculated using the χ^2^ test. ^**^Total number does not include missing data.

### 3.2 Motive for choosing to work in the healthcare sector and the individual performance of medical doctors

Table 2 shows the rates and the percentage distribution of the levels of work satisfaction based on responses to questions about individual performance driven by individual motivation (using the Kruskal-Wallis test) to assess differences in the proportion of ordinal scale variables between three groups of doctors. The feeling of having a valuable job and the sense of responsibility for the outcomes provided significantly greater satisfaction for medical doctors whose main motive was to help patients, compared to other doctors who opted for the “nature of the work itself” or “other” reasons. Doctors whose main motive for choosing the profession was to help patients are more likely to feel high satisfaction with “The notion that my work is valuable in itself” than those motivated by other factors (42.5% vs. 27.0% for “other” and 28.3% for “Nature of the work itself”), as well as with “The sense of responsibility for the outcomes” (50.0% vs. 35.1% for “other” and 36.3% for “Nature of the work itself”). Doctors driven by “other” motives derived the least satisfaction from these two factors. The recovery of patients was the greatest source of satisfaction for all doctors. However, it is worth noting that doctors motivated by “other” motives, rather than “helping patients” and “nature of the work itself,” reported lower satisfaction with the aspects presented in the table.

**Table 2:** Impact of individual motivation (the reason for choosing to work in the healthcare sector) on individual performance.

**Motive for choosing to work in the healthcare sector**	**High satisfaction**	**Moderate satisfaction**	**Moderate dissatisfaction**	**High dissatisfaction**
**Planning own work** ***p*** = **0.289**				
Helping patients	16.3% (13)	46.3% (37)	28.8% (23)	8.8% (7)
Nature of the work itself	10.9% (10)	41.3% (38)	35.9% (33)	12.0% (11)
Other	21.6% (8)	27.0% (10)	35.1% (13)	16.2% (6)
*In general*	***14.8% (31)***	***40.7% (85)***	***33.0% (69)***	***11.5% (24)***
^*^The notion that my work is valuable in itself ***p** =* **0.003**				
Helping patients	42.5% (34)	48.8% (39)	7.5% (6)	1.3% (1)
Nature of the work itself	28.3% (26)	53.3% (49)	15.2% (14)	3.3% (3)
Other	27.0% (10)	37.8% (14)	16.2% (6)	18.9% (7)
*In general*	***33.5% (70)***	***48.8% (102)***	***12.4% (26)***	***5.3% (11)***
^*^The sense of responsibility for the outcomes ***p** =* **0.024**				
Helping patients	50.0% (40)	46.3% (37)	1.3% (1)	2.5 %(2)
Nature of the work itself	36.3% (33)	48.4% (44)	11.0% (10)	4.4% (4)
Other	35.1% (13)	43.2% (16)	13.5% (5)	8.1% (3)
*In general*	***41.3% (86)***	***46.6% (97)***	***7.7% (16)***	***4.3% (9)***
**Recovery of patients** ***p*** = **0.289**				
Helping patients	42.5% (34)	53.8% (43)	3.8% (3)	0.0% (0)
Nature of the work itself	34.8% (32)	57.6% (53)	7.6% (7)	0.0% (0)
Other	36.1% (13)	52.8% (19)	5.6% (2)	5.6% (2)
*In general*	***38.0% (79)***	***55.3% (115)***	***5.8% (12)***	***1.0% (2)***
**Setting your own goals** ***p*** = **0.130**				
Helping patients	16.5% (13)	63.3% (50)	15.2% (12)	5.1% (4)
Nature of the work itself	4.4% (4)	63.7% (58)	26.4% (24)	5.5% (5)
Other	22.2% (8)	44.4% (16)	27.8% (10)	5.6% (2)
*In general*	***12.1% (25)***	***60.2% (124)***	***22.3% (46)***	***5.3% (11)***

^*^Significance (*p* < 0.05) determined using the Kruskal-Wallis test.

These aspects of individual performance were the least satisfying for doctors who chose the “other” option. This means there is a relationship between individual motivation and the individual performance of medical doctors, as measured by “the notion that my work is valuable in itself” and “responsibility for the outcomes.” This finding confirms the study's hypothesis as far as the individual performance is tested (measured) using these two questions.

## 4 Discussion

This study confirms that there is a link between the motives for choosing to work in the healthcare sector and the individual performance of medical doctors.

As a rule, people do not apply for jobs if they lack specific skills. Other studies show that individuals are motivated to choose and pursue a specific career path as long as they believe they will succeed in the position (37, 38). In this study, 46% of the respondents mentioned that the main motives for choosing to work in the healthcare sector were the nature of the work itself, or the value of the work itself, as defined in this study. Thirty-seven percent of the surveyed medical doctors mentioned “helping patients” as the main reason for choosing their profession. Only 16.6% of the doctors surveyed explained that they decided to work as medical professionals for reasons “other” than their interest in medicine or a humanitarian motive. Although the literature data are inconsistent, the results of this study largely overlap with the conclusions drawn by Gasiorowski in a two-stage study published in 2015. This study was conducted among medical students at the end of the their first year of medical school (*n* = 143) and in the 6^th^ year of their studies (*n* = 119) at the Pomeranian Medical University. The same questionnaire was used at both stages. The surveys showed that intrinsic motives, such as “willingness to help others,” followed by “interest in medicine,” were the most common reasons for choosing medical studies among 1^st^-year students (39). Interestingly, these motives have changed among male students in their 6^th^ year of medical studies. Altruistic motivation ranked the second, with most respondents feeling more motivated by scientific reasons, such as “interest in medical knowledge and research.” Puljak et al. demonstrated the precedence of research-related motives, which were followed by humanitarian rationale (40). Similar results were reported in a 2017 study of 123 Japanese doctors (41), where the scientific motive was ranked first, followed by the desire to improve community health. This analysis included only female physicians due to the low percentage of women employed in this profession in Japan. In a study on medical students, ML Crossley and A. Mubarik identified the following order of priority for choosing medical studies: professional career, patient care, use of personal skills, and interest in science (42). Altruism and scientific challenges were the main motives for medical students in a 2006 study by McManus et al. (43). In a study by Gaspar et al. (44), 89% of the family doctors surveyed (*n* = 109) argued that their career choices were driven by intrinsic motivation. Of these respondents, 33.9% and 59.6% reported feeling strongly or moderately motivated, respectively. On the other hand, referring to the results of the research on the group of aspects identified in our analysis as “other”—which had the relatively lowest percentage share—recognition and personal life (i.e., the standard of living that can be provided for the family through work) were most frequently mentioned. These were followed by job security/employment stability, salary, and personal development. When comparing these findings with analyses previously conducted by other researchers, an asymmetry is noticeable—an inverse hierarchy of values. Social and professional status were the main reasons for choosing the medical profession in the study by Dastjerdi et al. (45). These studies analyzed the direct reasons for choosing the medical profession, but included medical students as respondents, which differs from the population in this study, which focuses on professionally active medical doctors. Heikkilä et al. (29) surveyed a sample of 7,758 Finnish doctors, who were asked about their reasons for choosing their profession. Five factors were mentioned: a good place to work, defined as a place where doctors receive support from colleagues and superiors; career and professional development; and non-work-related aspects such as personal contacts, personal reputation, and finances. This study revealed significant differences among the surveyed doctors, especially between female and male doctors. Compared to male doctors, female doctors were more interested in the quality of education, career development, and lifestyle. They also showed less interest than men in the financial aspects of their profession. Moreover, the importance of career and professional development as a motivation decreased with age. In 2016, Winter and Thaler analyzed the reasons for choosing the medical profession using a sample *(n* = 563) of medical students in Germany. The data indicate that the career choices were influenced by both other-directed motives, such as altruism and working for the benefit of the society, as well as self-focused motives, such as financial security and work-life balance (46).

The study also analyzes the relationship between the reasons medical doctors choose to work in the healthcare sector and their individual performance.

In the 1940s and 1950s, psychologist Abraham Maslow argued that human motivation and personal development were directly interrelated, particularly in individuals who operated at the highest possible level while seeking fulfillment of their needs (47). This study demonstrates that medical doctors whose primary reason for choosing a career in the healthcare sector was to help patients derived more satisfaction from aspects related to individual performance, such as the “notion that my work is valuable in itself” and “responsibility for the outcomes.” These factors were the least satisfying for medical doctors who opted for the “other” reasons. Few studies analyze the relationship between the reasons for choosing the medical profession and the individual performance at work. In 2011, Rolfe et al. (48) examined doctors over a 16-year period following graduation from a medical university in Australia and found no differences between the study groups in terms of academic achievements, number of scientific papers published, or their practice or professional careers based on the motivation for choosing the medical profession. A study by Prytherch et al. (49), which included neonatologists working in rural areas of Tanzania, yielded results consistent with the conclusions of the present study and other referenced studies. Notwithstanding the difficulties with defining intrinsic motivation, this study concluded that those who were intrinsically motivated showed continuous readiness to give their best at work.

## 5 Strengths and limitation of the study

The random selection and relatively robust sample size are the strengths of this study. It also relies on the WHO questionnaire and incorporates the relevant theory as the study framework and to explain the study findings. The results of this analysis, along with and other research carried out in both developed and developing countries, were compared to highlight that the complex problems discussed are universal and faced by healthcare systems worldwide. It is also evident that more research is needed to include hospitals in small-town settings.

## 6 Conclusions and practical implications

The following conclusions and implications can be formulated based on this analysis:

1. Physicians whose primary reason for choosing a career in the healthcare sector was the desire to help patients experienced greater satisfaction with aspects related to individual performance, such as the “notion that my work is valuable in itself” and “responsibility for the outcomes.” Thus, individual motivation impacts the individual performance of medical doctors when measured by these aspects.
2. Job satisfaction, as related to performance aspects, was significantly lower among medical doctors who chose to work in the healthcare sector for reasons other than the desire to help patients or taking an interest in medicine (humanitarian and scientific rationale).
3. The research provides information on areas in need of corrective action at three levels: the individual, the workplace department/public hospital, and the system. This will help lay the groundwork for improving physician motivation and, consequently, performance, especially on an individual basis:

a) The distinctive features of this profession, including personal qualities, should perhaps be incorporated into the admission criteria for medical studies. It is also advised to consider organizing career counseling for graduates to raise awareness of the desired personal prerequisites and individual characteristics, including potential risks such as susceptibility to professional burnout, as well as establishing dedicated psychological clinics for working doctors.
b) It seems that in the development of programs aimed at improving organizational functioning, particularly in terms of occupational hygiene—i.e., external factors such as working conditions, payment, or policies—the involvement of doctors with “other” reasons for working can play an important role.
c) Regarding physicians who are convinced that their work is valuable and who evaluate their individual effectiveness significantly better, the study's findings may suggest the need to increase physicians' decision-making autonomy.
d) Managers should be prepared to study employee motivation to avoid underestimating the importance and impact of occupational choice motivation on efficiency and to apply a differentiated incentive system for employees.

## Data Availability

The raw data supporting the conclusions of this article will be made available by the authors, without undue reservation.
